# Online Health Information Seeking by Individuals With Physical Disabilities Caused by Neurological Conditions in Saudi Arabia

**DOI:** 10.7759/cureus.34460

**Published:** 2023-01-31

**Authors:** Sarah S Aldharman, Mohammed K Althagafi, Abdulaziz A Alzahrani, Leinah H Alshahrani, Mohammad O Abu Zahirah, Amnah S Alharthi, Abdullah Madkhali, Zainah Al-Qahtani

**Affiliations:** 1 College of Medicine, King Saud Bin Abdulaziz University for Health Sciences, Riyadh, SAU; 2 College of Medicine, King Abdulaziz University, Jeddah, SAU; 3 College of Medicine, King Khalid University, Abha, SAU; 4 Neurology, King Faisal Hospital, Makkah, SAU; 5 College of Medicine, Jazan University, Jazan, SAU; 6 Department of Internal Medicine, King Khalid University, Abha, SAU

**Keywords:** online health information seeking, saudi arabia, neurological diseases, disabilities, information

## Abstract

Background

In recent years, there has been an increase in the use of the internet and information technology for accessing health information. This study aimed to determine the factors that affect patients with neurological disabilities and their willingness to search for information via the internet. In addition, we aimed to assess how patients manage this information, considering the increasing availability of online information and websites that discuss health and diseases, as well as the spread of communication technology and its accessibility to the public.

Methodology

A cross-sectional, online, self-administered, questionnaire study was conducted in Saudi Arabia. The study targeted patients with neurological diseases who had disabilities. The questionnaire was designed to measure the demographic data, physical disability using the 10-item physical function component of the 36-Item Short Form Health survey, the perceived usefulness of online health information, the perceived ease of use, and the perceived risk of online health information. Lastly, the questionnaire measured online health information-seeking intentions and information use. Data analysis was performed using RStudio (R version 4.1.1, Posit, Boston, USA).

Results

We received 1,179 responses, of which 399 were excluded due to using another way to get information rather than the internet, 31 did not have neurological disabilities, and 136 did not complete the questionnaire. The remaining 613 responses were included in the final analysis. The participants were mostly male (54.6%), not married (54.6%), and had a bachelor’s degree (49.99%). The average age of participants was 18-25 years (24.5%) and 26-35 years (23.2%), Additionally, most participants resided in the western (26.9%) and eastern (25.9%) regions. Most participants (39.5%) had a monthly income of 5,000 to 10,000 SAR. Further, the most common neurological diseases were multiple sclerosis and epilepsy (26.9% and 23.2%, respectively). Based on the analysis of the data, the most important factor affecting online health information-seeking intention was that people with higher monthly incomes were more likely to seek online health information; these included people with an income of 10,000-20,000 SAR and >20,000 SAR. The most common factor affecting information use was the region of residence. The southern and western regions were less likely to adopt information use.

Conclusions

The monthly income and the area of residence had the greatest impact on people with neurological disabilities who sought online health information in the Kingdom of Saudi Arabia. Educational campaigns and workshops should be arranged to increase the population’s awareness of this topic, as well as to reveal the extent and prevalence of online health information seeking among disabled patients.

## Introduction

The use of the internet and information technology for seeking health information has been increasing in recent years [1,2]. The Google platform is one of the most frequently used tools for seeking information that includes healthcare awareness [2]. Patients with physical disabilities believe that the internet is a valuable tool for seeking information regarding their conditions due to mobility challenges in receiving information from the clinic and their restricted physical activity [3]. Neurological conditions affect both mental and physical functions [3]. Our aim in this study is to understand the factors related to disabled people seeking information via the internet and how they manage it. Previous literature claims that there is an increase in the number of patients seeking input from the internet concerning their neurological diseases, including meningitis, Alzheimer’s disease, epilepsy, and various central nervous system (CNS) conditions [4-9]. A report published by the World Health Organization on December 14, 2011, showed that 1 billion people suffered from some type of disability, i.e., around 15% in 2010 compared to 10% in 1970, and found that the biggest factor affecting this ratio is chronic health diseases, i.e., around 66.5% of all age groups [10]. In Saudi Arabia, a 2018 study reported that one in every 30 persons has some form of disability, and this ratio increased in people aged 60 years, males, and those living in Tabuk, Madinah, and the Northern Border regions [11].

Cognitive perceptions can be used to predict the online health information usage and seeking behavior of neurologically impaired persons. The increased impairment level of people influences how often they search for health information online, but it has no bearing on how they use the acquired information. Furthermore, obtaining more online health information does not lead to a person using it more frequently, indicating that these two activities should be carefully distinguished from one another [3]. We have calculated the additional long-term health issues that persons with co-occurring autism and intellectual impairments face, which is more than a twofold disadvantage. Staff members working with these individuals face difficulties due to the complexity of assessments, diagnoses, and treatments for additional medical conditions. In this situation, sensory impairments and mental health issues, in particular, compound the person’s pre-existing communication and cognitive issues. Planning is crucial, and employees must be properly taught, equipped, resourced, and ready to handle the difficulty of working with individuals who have these problems [12]. The prevalence of physical multi-morbidity among people with an intellectual disability is so great that connections with mental illness are not observed. The whole community of people with intellectual impairments needs mental health treatments and preventative measures, and subgroups should not be targeted primarily on the burden of their overall health [13]. A study was conducted regarding the internet health information usage behavior of individuals with physical disabilities. Data were collected from 243 participants. The results showed that online usage of health information increases the perception of benefits while decreasing risk perceptions. Moreover, they found that the accuracy, completeness, currency, and transparency of online health information anticipate information quality [14]. Few studies have been focused on this topic, and it is considered a gap that needs to be filled. Further, the internet can be viewed as a source of misleading information that affects patient health and leads to serious complications in patients who follow information from the internet [3]. This study aimed to investigate how neurologically disabled patients search and use online health information and assess related factors in Saudi Arabia.

## Materials and methods

This is a descriptive, cross-sectional, questionnaire-based study conducted in Saudi Arabia between September 2022 and November 2022. The target population was individuals with physical disabilities due to neurological conditions from different areas of Saudi Arabia. The data were gathered using a self-administered questionnaire which was distributed electronically via Google Forms. The data were uploaded into Microsoft Excel, and RStudio (R version 4.1.1, Posit, Boston, USA) was utilized to analyze data.

Study population

Our target population of this study was individuals with physical disabilities due to neurological conditions (e.g., multiple sclerosis, amyotrophic lateral sclerosis, Parkinson’s disease, muscular dystrophy, Alzheimer’s disease, stroke, benign brain tumors, epilepsy, myasthenia gravis, motor neuron disorders, and other neurological conditions) who were aged at least 18 from different regions of Saudi Arabia.

Inclusion and exclusion criteria

Individuals of both genders aged ≥18 years old with certain physical impairments caused by neurological problems in Saudi Arabia were included in the study. Participants who did not have neurological diseases, did not search health information online for their condition, answered “No” to all questions of the first part (the disability part), did not complete the entire questionnaire, or did not consent to participate were excluded from the study. Moreover, we provided a question in the beginning “Do you have any cognitive problems that might impede your understanding of the questions?.” Those who answered “Yes” were excluded from the study.

Sampling and sample size estimation

The sample size was calculated using the OpenEpi® version 3.0 program (Centers for Disease Control and Prevention, USA). The required representative sample size was 385, with a margin of error of 5%, a confidence level of 95%, and a population size of Saudi Arabia determined as 3,400,000. We planned to exceed the expected sample size to overcome any potential omissions. Non-probability consecutive sampling approach was employed.

Data collection tool and procedure

A validated and reliable questionnaire from a previous study was used [3]. The questionnaire contains seven sections. The first section contains the demographic data of the participants (age, gender, etc.). The second section measures physical disability. The physical disability level was assessed utilizing the 10-item physical function component of the 36-Item Short Form Health (SF-36) survey [15]. Each item assesses a person’s ability to undertake a certain physical activity. Considering the SF-36 scoring guide [16], a physical function score was computed by averaging the 10-item scores. The possible responses were 1 = Yes, limited a lot; 2 = Yes, limited a little; and 3 = No, not limited at all. The range of this score is 0-100, with higher scores representing better physical functions. We subtracted this value from 100 to generate a disability score, with larger disability scores representing more severe impairments. The third section is a four-item scale that measured the perceived usefulness of online health information. The fourth section had four items that measure perceived ease of use. The fifth section also contained four items regarding the perceived risk of online health information. The sixth section measured online health information-seeking (OHIS) intention using three items. The final section examined information use via three items. From sections three to seven, the items were evaluated on a seven-point Likert scale (1, strongly disagree; 2, disagree; 3, slightly disagree; 4, neutral; 5, slightly agree; 6, agree; 7, strongly agree).

Electronic Google Forms were used and distributed on different social media platforms, such as WhatsApp, Twitter, and Telegram. All information was kept anonymous, and participation in the study was optional with the first page designated to obtain consent. Ethical approval of the study was obtained from the Research Ethics Committee at King Khalid University (reference number: ECM#2022-2403).

Data management and statistical analysis

After distributing the questionnaire, they were checked for completeness and any missing information. The online data gathering system ensured that all items must be answered, ensuring that no incomplete surveys would be submitted. Subsequently, the data were entered into Microsoft Excel and transferred to RStudio for further analysis. Data analysis was performed in RStudio. Categorical data were presented as frequencies and percentages, while numerical data were expressed as mean ± SD. A Pearson’s correlation test was used to assess the bivariate correlations between different domains in a correlation matrix. To assess the independent factors that were associated with OHIS intention and use of information, we constructed two hierarchical regression models using each outcome as a dependent variable in a separate model. Each model consisted of three steps based on the independent variables as follows: step 1 contained the sociodemographic variables, step 2 contained sociodemographic variables and disability, and step 3 contained sociodemographic variables, disability, perceived usefulness, perceived ease of use, and the perceived risk. The average of domain items was used for the hierarchical regression. The results of the regression analysis were expressed as beta coefficients and 95% confidence intervals (95% CIs). A p-value <0.05 was used to indicate statistical significance.

## Results

Evaluation of measures

Initially, the questionnaire consisted of 37 items and eight domains, including patients’ medical history (three items), sociodemographic characteristics (six items), disability (10 items), perceived usefulness (four items), perceived ease of use (four items), perceived risk (four items), information use (three items), and intention (three items). However, based on an exploratory factor analysis using a Promax rotation method, we excluded five items because they were not significantly loaded to their respective domain (factor loading <0.50). These items included one item from the disability domain, two items from the perceived ease of use domain, one item from the perceived risk domain, and one item from the intention domain (Appendices).

Subsequently, we conducted a confirmatory factor analysis to ascertain factor loadings. As demonstrated in Table 1, the factor loadings of all items were above 0.50. Additionally, the domains showed good internal consistency measures (Cronbach’s alpha ranging between 0.835 and 0.920) and composite reliability indicators (0.845 to 0.921), and the average variance extracted (AVE) values were all above 0.50. The results of the confirmatory factor analysis are shown in Table 1. The discriminant validity of the model was further confirmed by the fact that the square roots of AVE were higher than the correlation coefficients between different domains, as shown in Table 2.

**Table 1:** Results of the confirmatory factor analysis. AVE = average variance extracted; CR = composite reliability; Crα = Cronbach’s alpha; SFL = standardized factor loadings

Parameter	SFL	AVE	CR	Crα
Disability		0.556	0.881	0.881
Vigorous activities, such as running, lifting heavy objects, participating in strenuous sports	0.584			
Moderate activities, such as moving a table, pushing a vacuum cleaner, bowling, or playing golf	0.669			
Lifting or carrying groceries	0.623			
Climbing several flights of stairs	0.793			
Bending, kneeling, or stooping	0.754			
Walking more than one mile	0.722			
Walking several blocks	0.777			
Walking one block	0.559			
Bathing or dressing yourself	0.539			
Perceived usefulness		0.744	0.921	0.920
Online health information improves my capability of managing my conditions	0.863			
Online health information increases my knowledge of my personal health	0.889			
Online health information helps me relieve stress over my new symptoms or drug side effects	0.820			
I find online health information to be useful	0.876			
Information use		0.718	0.883	0.877
I follow the advice offered by online health information	0.878			
Online health information heavily influences my personal health decisions	0.908			
I use online health information to cope with my emotions such as fear, stress, and frustration	0.747			
Risk		0.648	0.845	0.835
Online health information could be misleading	0.832			
Online health information could harm my health	0.896			
I could be stressed out due to exaggerating online health information	0.670			
Perceived ease of use		0.805	0.892	0.891
I find it easy to search the internet for health information	0.916			
I find it easy to get health websites to return what I want	0.878			
Intention		0.809	0.894	0.893
I intend to continue seeking health information from the internet	0.932			
I plan to continue using the internet to obtain health information	0.866			

**Table 2:** A correlation matrix showing the correlation between different domains. AVE = average variance extracted; SD = standard deviation

	1	2	3	4	5	6
1. Disability	1					
2. Perceived usefulness	-0.06	1				
3. Ease of use	-0.03	0.68	1			
4. Risk	0.00	0.4	0.36	1		
5. intention	-0.06	0.74	0.63	0.43	1	
6. Information use	0.02	0.61	0.61	0.37	0.66	1
AVE	0.556	0.744	0.805	0.648	0.809	0.718
Square root of AVE	0.746	0.863	0.897	0.805	0.899	0.847
Mean	1.412	4.465	4.276	4.640	4.356	4.070
SD	0.350	1.535	1.644	1.416	1.550	1.519

Sociodemographic characteristics

**Figure 1:**
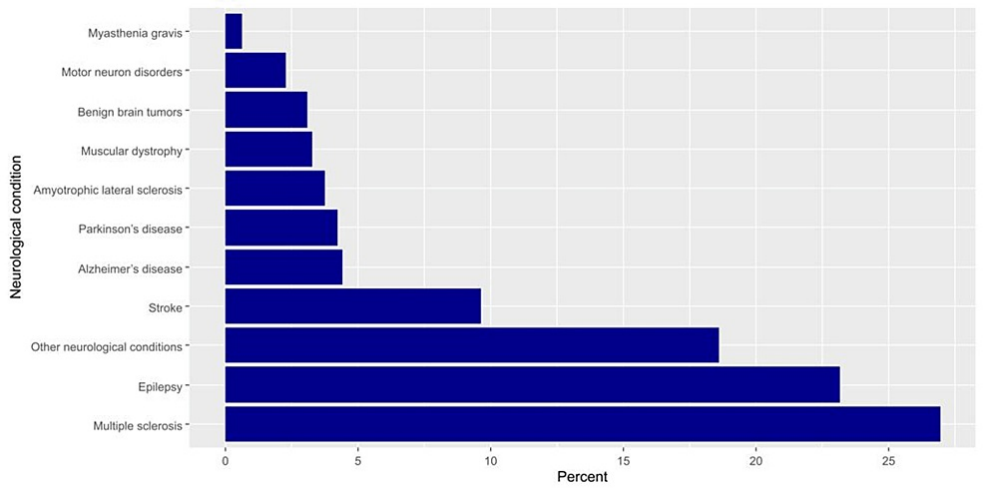
The percentages of neurological conditions among the participants.

We received responses from 1,179 participants on the online platform. However, we excluded the responses from 399 participants who did not use the internet to get information about the condition, 31 patients without neurological conditions, and 136 patients with missing responses. Therefore, we ultimately analyzed the responses of 613 participants. More than half of the participants were males (54.6%), whereas less than half were married (45.4%) and had attained a bachelor’s degree (49.9%). The most common age categories were 18-25 years (24.5%) and 26-35 years (23.2%), and the most common regions of residence were the western and eastern regions (26.9% and 25.9%, respectively). Additionally, 39.0% of the respondents had a monthly income of 5,000-10,000 SAR. Detailed sociodemographic characteristics of the participants are shown in Table 3. The most common neurological conditions among the participants were multiple sclerosis (26.9%) and epilepsy (23.2%). Figure 1 illustrates the percentages of neurological conditions among the participants.

**Table 3:** Sociodemographic characteristics of the participants (n = 613).

Parameter	Category	N (%)
Gender	Male	335 (54.6%)
	Female	278 (45.4%)
Age (years)	<18	36 (5.9%)
	18–25	150 (24.5%)
	26–35	142 (23.2%)
	36–45	113 (18.4%)
	46–55	85 (13.9%)
	>55	87 (14.2%)
Educational level	General education	289 (47.1%)
	Bachelor	306 (49.9%)
	Post-graduate	18 (2.9%)
Marital status	Single	223 (36.4%)
	Married	278 (45.4%)
	Divorced	71 (11.6%)
	Widow	41 (6.7%)
Monthly income (Saudi Riyals)	<5,000	102 (16.6%)
	5,000–10,000	239 (39.0%)
	10,000–20,000	160 (26.1%)
	>20,000	112 (18.3%)
Region	Northern	38 (6.2%)
	Southern	157 (25.6%)
	Eastern	159 (25.9%)
	Western	165 (26.9%)
	Central	94 (15.3%)

Factors affecting online health information-seeking intention

Based on the hierarchical regression analysis, participants with high monthly incomes were more likely to intend to seek online health information; these included an income of 10,000-20,000 SAR (β = 0.59, 95% CI = 0.17 to 1.01; p = 0.006) and >20,000 SAR (β = 0.75, 95% CI = 0.33 to 1.18; p < 0.001). Similarly, both the monthly income categories were significant predictors of OHIS intention (β = 0.58, 95% CI = 0.16 to 1.00; p = 0.007 for 10,000-20,000 SAR, and β = 0.71, 95% CI = 0.28 to 1.14; p = 0.001 for >20,000 SAR); however, disability did not predict the outcome variable. In step 3, OHIS intention was independently associated with the following variables of online information: the perceived usefulness (β = 0.52, 95% CI = 0.44 to 0.59; p < 0.001), perceived ease of use (β = 0.21, 95% CI = 0.15 to 0.28; p < 0.001), and perceived risks (β = 0.15, 95% CI = 0.09 to 0.22; p < 0.001). Table 4 shows the outcomes of the hierarchical regression analysis for OHIS intention.

**Table 4:** Outcomes of the hierarchical regression analysis for online health information-seeking intention.

Parameter	Category	Step 1		Step 2		Step 3	
		Beta (95% CI)	P-value	Beta (95% CI)	P-value	Beta (95% CI)	P-value
Gender	Male	—		—		—	
	Female	0.13 (-0.12 to 0.37)	0.318	0.13 (-0.12 to 0.38)	0.297	0.03 (-0.13 to 0.19)	0.717
Age (years)	<18	—		—		—	
	18–25	0.13 (-0.43 to 0.70)	0.639	0.12 (-0.44 to 0.68)	0.681	0.26 (-0.11 to 0.64)	0.168
	26–35	0.39 (-0.21 to 0.98)	0.203	0.37 (-0.23 to 0.97)	0.223	0.29 (-0.11 to 0.69)	0.154
	36–45	0.34 (-0.29 to 0.97)	0.289	0.36 (-0.27 to 0.98)	0.266	0.39 (-0.03 to 0.81)	0.067
	46–55	-0.29 (-0.95 to 0.38)	0.402	-0.22 (-0.89 to 0.46)	0.528	0.09 (-0.36 to 0.54)	0.697
	>55	-0.65 (-1.31 to 0.00)	0.052	-0.61 (-1.27 to 0.04)	0.067	0.18 (-0.26 to 0.62)	0.425
Educational level	General education	—		—		—	
	Bachelor	0.22 (-0.04 to 0.48)	0.095	0.21 (-0.05 to 0.47)	0.116	0.10 (-0.08 to 0.27)	0.289
	Post-graduate	-0.65 (-1.40 to 0.10)	0.088	-0.67 (-1.42 to 0.08)	0.079	-0.15 (-0.65 to 0.35)	0.565
Marital status	Single	—		—		—	
	Married	-0.10 (-0.44 to 0.23)	0.543	-0.10 (-0.44 to 0.23)	0.546	0.06 (-0.16 to 0.29)	0.57
	Divorced	-0.45 (-0.92 to 0.03)	0.066	-0.46 (-0.94 to 0.02)	0.058	0.02 (-0.31 to 0.34)	0.905
	Widow	0.34 (-0.26 to 0.95)	0.267	0.40 (-0.21 to 1.02)	0.196	0.13 (-0.27 to 0.54)	0.519
Monthly income (Saudi Riyals)	<5,000	—		—		—	
	5,000–10,000	0.20 (-0.17 to 0.57)	0.294	0.18 (-0.18 to 0.55)	0.327	0.03 (-0.22 to 0.27)	0.838
	10,000–20,000	0.59 (0.17 to 1.01)	0.006	0.58 (0.16 to 1.00)	0.007	0.09 (-0.19 to 0.37)	0.527
	>20,000	0.75 (0.33 to 1.18)	<0.001	0.71 (0.28 to 1.14)	0.001	0.27 (-0.02 to 0.55)	0.071
Region	Northern	—		—		—	
	Southern	-0.39 (-0.93 to 0.16)	0.165	-0.42 (-0.97 to 0.13)	0.133	-0.04 (-0.40 to 0.33)	0.840
	Eastern	-0.07 (-0.61 to 0.46)	0.791	-0.01 (-0.54 to 0.53)	0.985	0.34 (-0.02 to 0.70)	0.064
	Western	0.04 (-0.50 to 0.58)	0.888	0.06 (-0.48 to 0.60)	0.828	0.08 (-0.28 to 0.44)	0.664
	Central	0.22 (-0.34 to 0.79)	0.440	0.28 (-0.28 to 0.85)	0.328	0.30 (-0.08 to 0.68)	0.121
Disability	Score	NA	NA	-0.32 (-0.70 to 0.06)	0.094	-0.16 (-0.41 to 0.09)	0.210
Perceived usefulness	Score	NA	NA	NA	NA	0.52 (0.44 to 0.59)	<0.001
Perceived ease of use	Score	NA	NA	NA	NA	0.21 (0.15 to 0.28)	<0.001
Risk	Score	NA	NA	NA	NA	0.15 (0.09 to 0.22)	<0.001
Model parameters	R^2^	0.119		0.123		0.615	
	Adjusted R^2^	0.092		0.095		0.601	
	R^2^ Change	0.119		0.004		0.492	

Factors affecting information use

Results of the second regression model showed that high monthly income categories were independently associated with information use in step 1 (β = 0.44, 95% CI = 0.02 to 0.86; p = 0.039 for 10,000-20,000 SAR, and β = 0.46, 95% CI = 0.04 to 0.89; p = 0.033 for >20,000 SAR) and in step 2 after the adjustment of disability (β = 0.44, 95% CI = 0.02 to 0.85; p = 0.041 for 10,000-20,000 SAR, and β = 0.45, 95% CI = 0.03 to 0.88, p = 0.038 for >20,000 SAR). However, monthly income did not influence information use in step 3. The region of residence was also a significant predictor of information use. Considering residence in the northern region as a reference group, participants residing in the southern and western regions were less likely to adopt information use in step 1 (β = -0.64, 95% CI = -1.19 to -0.10; p = 0.021, and β = -0.55, 95% CI = -1.08 to -0.02, p = 0.044, respectively) and step 2 (β = -0.65, 95% CI = -1.19 to -0.10; p = 0.020 and β = -0.55, 95% CI = -1.08 to -0.01; p = 0.046, respectively). However, residence in the western region was the sole predictor of regional differences associated with a lower likelihood of information use in step 3 (β = -0.53, 95% CI = -0.93 to -0.13; p = 0.010). Other independent predictors of information use included the perceived usefulness (β = 0.30, 95% CI = 0.22 to 0.39; p < 0.001), perceived ease of use (β = 0.33, 95% CI = 0.25 to 0.40; p < 0.001), and the perceived risks (β = 0.16, 95% CI = 0.08 to 0.23; p < 0.001). The outcomes of the hierarchical regression analysis for information use are shown in Table 5.

**Table 5:** Outcomes of the hierarchical regression analysis for information use.

Parameter	Category	Step 1		Step 2		Step 3	
		Beta (95% CI)	P-value	Beta (95% CI)	P-value	Beta (95% CI)	P-value
Gender	Male	—		—		—	
	Female	0.18 (-0.06 to 0.43)	0.143	0.18 (-0.06 to 0.43)	0.142	0.08 (-0.10 to 0.27)	0.365
Age (years)	<18	—		—		—	
	18 to 25	-0.28 (-0.83 to 0.28)	0.330	-0.28 (-0.84 to 0.28)	0.325	-0.17 (-0.58 to 0.25)	0.440
	26 to 35	0.07 (-0.52 to 0.66)	0.816	0.07 (-0.53 to 0.66)	0.825	0.06 (-0.38 to 0.51)	0.778
	36 to 45	-0.07 (-0.70 to 0.55)	0.822	-0.07 (-0.69 to 0.56)	0.830	-0.02 (-0.49 to 0.44)	0.918
	46 to 55	-0.23 (-0.89 to 0.44)	0.504	-0.21 (-0.88 to 0.46)	0.533	0.03 (-0.47 to 0.54)	0.892
	>55	-0.62 (-1.27 to 0.03)	0.063	-0.61 (-1.27 to 0.04)	0.067	0.07 (-0.43 to 0.56)	0.796
Educational level	General education	—		—		—	
	Bachelor	0.23 (-0.03 to 0.49)	0.083	0.23 (-0.03 to 0.49)	0.087	0.14 (-0.06 to 0.33)	0.178
	Post-graduate	-0.72 (-1.46 to 0.02)	0.057	-0.73 (-1.47 to 0.02)	0.056	-0.21 (-0.77 to 0.36)	0.474
Marital status	Single	—		—		—	
	Married	-0.10 (-0.44 to 0.23)	0.548	-0.10 (-0.44 to 0.23)	0.549	0.07 (-0.18 to 0.32)	0.581
	Divorced	-0.39 (-0.87 to 0.08)	0.103	-0.40 (-0.87 to 0.08)	0.101	0.07 (-0.30 to 0.44)	0.706
	Widow	0.20 (-0.41 to 0.80)	0.520	0.21 (-0.40 to 0.82)	0.499	0.04 (-0.42 to 0.50)	0.869
Monthly income (Saudi Riyal)	<5,000	—		—		—	
	5,000–10,000	0.14 (-0.22 to 0.51)	0.441	0.14 (-0.23 to 0.51)	0.450	-0.04 (-0.32 to 0.24)	0.777
	10,000–20,000	0.44 (0.02 to 0.86)	0.039	0.44 (0.02 to 0.85)	0.041	-0.01 (-0.32 to 0.31)	0.975
	>20,000	0.46 (0.04 to 0.89)	0.033	0.45 (0.03 to 0.88)	0.038	0.05 (-0.28 to 0.37)	0.783
Region	Northern	—		—		—	
	Southern	-0.64 (-1.19 to -0.10)	0.021	-0.65 (-1.19 to -0.10)	0.020	-0.30 (-0.71 to 0.11)	0.158
	Eastern	-0.31 (-0.84 to 0.22)	0.257	-0.29 (-0.83 to 0.24)	0.283	0.06 (-0.35 to 0.46)	0.790
	Western	-0.55 (-1.08 to -0.02)	0.044	-0.55 (-1.08 to -0.01)	0.046	-0.53 (-0.93 to -0.13)	0.010
	Central	0.17 (-0.39 to 0.73)	0.541	0.19 (-0.38 to 0.75)	0.517	0.22 (-0.21 to 0.64)	0.317
Disability	Score	NA	NA	-0.06 (-0.44 to 0.31)	0.745	0.06 (-0.22 to 0.34)	0.664
Perceived usefulness	Score	NA	NA	NA	NA	0.30 (0.22 to 0.39)	<0.001
Perceived ease of use	Score	NA	NA	NA	NA	0.33 (0.25 to 0.40)	<0.001
Risk	Score	NA	NA	NA	NA	0.16 (0.08 to 0.23)	<0.001
Model parameters	R^2^	0.093		0.094		0.495	
	Adjusted R^2^	0.066		0.065		0.476	
	R^2^ Change	0.094		0.0002		0.401	

## Discussion

This study aimed to assess factors related to online health-seeking among patients with physical disabilities due to neurological causes. The study included 613 participants, of whom more than half were males (54.6%), and nearly half had attained a bachelor’s degree (49.9%). Overall, 39.0% of the respondents had a monthly income of 5,000-10,000 SAR. The most common neurological conditions among the participants were multiple sclerosis (26.9%) and epilepsy (23.2%).

Considering factors that influence health-seeking behavior, and, in particular, online health seeking, monthly income was found to play a significant role as we discovered that participants with high monthly incomes were more likely to intend to seek online health information; these included an income of 10,000-20,000 SAR (p = 0.006) and >20,000 SAR (p < 0.001). In addition, OHIS intention was independently associated with perceived usefulness (p < 0.001), perceived ease of use (p < 0.001), and perceived risks (p < 0.001). These findings are consistent with another study with a sample size of 330, which showed an increase in an individual’s OHIS intention with raises in perceived usefulness, ease of use, and disability levels. The study revealed that OHIS intention was also anticipated by a negative relationship between perceived usefulness and disability, and a negative relationship between perceived usefulness and perceived ease of use, in contrast to a positive relationship between perceived ease of use and disability. It was also revealed that a person’s utilization of online health information is associated favorably with perceived usefulness and adversely with perceived risk, as well as the interplay between perceived usefulness and risk [3].

Sociodemographic factors also played a role as factors in information use prediction. High monthly income categories were independently associated with information use (p = 0.039). The region of residence was also another significant predictor of information use. Considering residence in the northern region as a reference group, participants residing in the southern and western regions were less likely to adopt information use, except in step 3, where residence in the western region was the sole predictor of regional differences associated with a lower likelihood of information use. A previous study reported few similarities to our findings, at a significance level ≤0.05, gender (p = 0.037), educational level (p < 0.0001), employment status (p = 0.000), besides monthly income (p < 0.0001) was found to be significantly associated with the use of the internet for health information purposes. The significance of income was justified by being employed, and earning more income ensures that individuals are capable of acquiring internet-ready devices such as smartphones and laptops [17].

Among factors that affect information use, according to our study, the female gender was insignificantly associated with online health-seeking behavior (p = 0.143). This was different from another study which showed a significant association between the female gender and online-seeking health information behaviors (p = 0.03) [18]. Research demonstrates that women are more likely than males to seek health-related information [19]. Another research illustrated that sociodemographic factors such as age and gender appear to be insignificantly related to information use (p = 0.76, p = 0.95, respectively), which is similar to our findings. This leaves us to acknowledge that there are disparities in the association between gender and OHIS behavior [20].

Further, education level showed no significant association in our study compared to other literature (p = 0.001) [18]. However, we found that there is no significant association between marital status and OHIS behavior. This finding is consistent with the other study which also showed no significant association between these two variables (p = 0.93), which demonstrates a good seeking discipline behavior whether individuals are married or not [18].

Individuals with physical disabilities caused by neurological problems consider whether to apply the health information depending on a logical analysis of the efficiency and risk of utilizing the information. Thus, despite the fact that the quality of internet health information may be questionable [21], individuals with neurological disabilities are not vulnerable victims to deceptive or incorrect information. This is compatible with a few reports of harm related to the implementation of internet health information [22].

It is important to worth noting that an individual’s disability level is not associated with the use of online health information. Although it seems that more impaired persons are more inclined to look for health information online, they are not more in favor to reveal their self-care and health-related decision-making than their counterparts with fewer disabilities.

In addition, according to McMullan [23], physicians may respond differently toward patients’ OHIS behaviors; they either feel intimidated and respond defensively or cooperate with patients to obtain and analyze the information and guide patients toward authentic and reliable health websites. It is obvious that a defensive response is unfavorable to patient-doctor rapport. Thus, doctors should also be informed about the variety of online health information and be able to evaluate the quality of online health information so they can better cooperate with their patients.

Overall, our results indicate that health portal accessibility for people with disabilities should get considerable attention from stakeholders to raise users’ perceptions of the usability of these websites. The accessibility of health information websites should be given full consideration, employing legal requirements, industry advocacy, and commercial advertisements [24].

With a limited number of studies published in this field, this study is considered a valuable base for evidence as it is the first to be performed in Saudi Arabia. Another strength of this study is that it included participants with a high response rate from variable backgrounds and socioeconomic status, which would aid the authorities in dealing with the issue from all aspects.

The findings of this study were limited by being a questionnaire-based study, which carries the potential of interviewer, recollection, and response bias, as well as issues of cost and funding, which, if available, would have been a crucial aid for targeting a larger population of the community, as well as detailed tackling of the study subject. We recommend that authorities and the Ministry of Health should construct and install technical assistance for medical purposes. Further, primary healthcare providers should recognize that patients are using the internet for health information and should be prepared to assist, encourage, motivate, and promote internet user skills among their patients.

## Conclusions

Individuals with physical disabilities due to neurological conditions with high monthly incomes were more likely to seek online health information. Perceived usefulness, perceived ease of use, and perceived risks were independently associated with OHIS intention. The area of residence was also independently associated with information use. Serial and frequent studies on the issue need to be conducted to generate more evidence and data regarding this topic.

## Appendices

**Table 6:** Description of the domains and items that were originally developed in the used survey. *: An asterisk indicates an excluded item

Domain	Item code	Item name
Disability	Disability_01	Vigorous activities, such as running, lifting heavy objects, participating in strenuous sports
	Disability_02	Moderate activities, such as moving a table, pushing a vacuum cleaner, bowling, or playing golf
	Disability_03	Lifting or carrying groceries
	Disability_04	Climbing several flights of stairs
	Disability_05	Climbing one flight of stairs*
	Disability_06	Bending, kneeling, or stooping
	Disability_07	Walking more than one mile
	Disability_08	Walking several blocks
	Disability_09	Walking one block
	Disability_10	Bathing or dressing yourself
Perceived usefulness	Use_01	Online health information improves my capability of managing my conditions
	Use_02	Online health information increases my knowledge of my personal health
	Use_03	Online health information helps me relieve stress over my new symptoms or drug side effects
	Use_04	I find online health information to be useful
Perceived ease of use	Ease_01	My interaction with health websites is clear and understandable*
	Ease_02	Seeking online health information does not require a lot of mental effort*
	Ease_03	I find it easy to search the Internet for health information
	Ease_04	I find it easy to get health websites to return what I want
Risk	Risk_01	Online health information could be misleading
	Risk_02	Online health information could harm my health
	Risk_03	I could make a wrong decision regarding my health based on poor-quality online health information*
	Risk_04	I could be stressed out due to exaggerating online health information
Information use	Info_01	I follow the advice offered by online health information
	Info_02	Online health information heavily influences my personal health decisions
	Info_03	I use online health information to cope with my emotions such as fear, stress, and frustration
Intention	Inten_01	I intend to continue seeking health information from the Internet
	Inten_02	I plan to continue using the Internet to obtain health information
	Inten_03	If I could, I would like to discontinue seeking online health information*
